# Six Novel Species of *Distoseptispora* (*Distoseptisporaceae*, *Distoseptisporales*) and *Helminthosporium* (*Massarinaceae*, *Pleosporales*) Isolated from Terrestrial Habitats in Southern China

**DOI:** 10.3390/jof11070494

**Published:** 2025-06-29

**Authors:** Ming-Gen Liao, Xing-Xing Luo, Ji-Wen Xia, Ya-Fen Hu, Xiu-Guo Zhang, Lian-Hu Zhang, Xian-Peng Zhang, Zhao-Huan Xu, Jian Ma

**Affiliations:** 1 College of Agronomy, Jiangxi Agricultural University, Nanchang 330045, China; kuutaa@163.com (M.-G.L.); luoxingxing99@126.com (X.-X.L.); hyf604325418@126.com (Y.-F.H.); zhanglianhu0328@163.com (L.-H.Z.); xpzhang@jxau.edu.cn (X.-P.Z.); hzzhaohuan@163.com (Z.-H.X.); 2 College of Agriculture and Forestry, Linyi University, Linyi 276300, China; xiajiwen@lyu.edu.cn; 3 Shandong Provincial Key Laboratory for Biology of Vegetable Diseases and Insect Pests, College of Plant Protection, Shandong Agricultural University, Tai’an 271018, China; zhxg@sdau.edu.cn; 4 Jiangxi Key Laboratory for Excavation and Utilization of Agricultural Microorganisms, Jiangxi Agricultural University, Nanchang 330045, China

**Keywords:** asexual *Ascomycota*, morphological characteristics, new species, phylogenetic analyses, terrestrial habitats, systematics

## Abstract

Saprobic hyphomycetous fungi exhibit high colonization density and diversity on rotting woody plant material. During our continuing mycological research in the forest ecosystem of Jiangxi, Fujian and Zhejiang provinces, China, several *Distoseptispora*-like and *Helminthosporium*-like strains were isolated from unidentified dead branches in terrestrial habitats. Based on morphological comparisons and multi-locus phylogenetic analyses using maximum-likelihood (ML) and Bayesian inference (BI), six novel species of *Distoseptispora* (*D. terrestris*, *D. wuyishanensis*, *D. zhejiangensis*) and *Helminthosporium* (*H. ganzhouense*, *H. jiangxiense*, *H. saprophyticum*) were introduced, and one known species, *H. velutinum* was reported. The species diversity within *Distoseptispora* and *Helminthosporium* was supplemented in this study.

## 1. Introduction

Fungi represent a critical component of forest ecosystems, functioning as decomposers, mutualists, or pathogens that significantly influence plant nutrition and carbon cycling within these systems [1]. The earliest conservative estimate by Hawksworth [2] suggested a global fungal species count of 1.5 million. Subsequently, DNA sequencing technologies have led to a shift in the estimated number of fungal species, from a range of 2.2 to 3.8 million based on host association to a range of 11.7 to 13.2 million based on high-throughput sequencing [3,4]. However, current scientific consensus estimates that only approximately 150,000 fungal species have been discovered and formally described [5,6].

The genus *Distoseptispora* K.D. Hyde, McKenzie & Maharachch. was established by Su et al. [7] based on molecular support and morphological characteristics. The majority of *Distoseptispora* species was described based on morphological characteristics of asexual morphs, and is mainly characterized by acrogenous, solitary, distoseptate or euseptate conidia seceding schizolytically from monoblastic or polyblastic, integrated, terminal, determinate or percurrently extending conidiogenous cells on macronematous, unbranched, septate conidiophores [8,9,10]. Only four species, *D. euseptata* W.L. Li, H.Y. Su & Jian K. Liu, *D. hyalina* Monkai & Phookamsak, *D. licualae* Konta & K.D. Hyde, and *D. suoluoensis* J. Yang, Maharachch. & K.D. Hyde were observed from their sexual morphs [11,12,13]. However, the teleomorph–anamorph relationship remains unverified in *D. euseptata* and *D. hyalina* due to lack of cultural studies and molecular data [11,12,14]. To date, 94 epithets for *Distoseptispora* are listed in Index Fungorum [15]. However, *D. submersa* Z.L. Luo, K.D. Hyde & H.Y. Su was synonymized with *D. tectonae* Doilom & K.D. Hyde by Dong et al. [16] based on minimal nucleotide divergence and high morphological similarity. Thus, *Distoseptispora* currently includes 93 valid taxa, and all species identified based on morphological and phylogenetic analyses.

The genus *Helminthosporium* Link was established by Link [17] with an asexual saprobe, *H. velutinum* Link as the type species, and is mainly characterized by distinct, unbranched or sparingly branched, determinate or percurrently extending conidiophores, and tretic, integrated, terminal or intercalary conidiogenous cells with small conspicuous pores beneath the septa, and solitary (rarely in short chains), acropleurogenous, usually obclavate, sometimes rostrate, distoseptate conidia frequently with a prominent, dark brown to black scar at the base [18,19,20]. Most of *Helminthosporium* species are described as asexual morph on dead branches and submerged wood, and only seven species, namely *H. massarinum* Kaz. Tanaka, K. Hiray. & Shirouzu, *H. microsorum* D. Sacc., *H. oligosporum* (Corda) S. Hughes, *H. puerense* L. Lu & Tibpromma, *H. quercicola* (M.E. Barr) Voglmayr & Jaklitsch, *H. quercinum* Voglmayr & Jaklitsch, and *H. tiliae* (Link) Fr., were proposed to be related massaria- or splachnonema-like teleomorphs [18,21]. To date, more than 780 epithets have been listed in *Helminthosporium* [15]. Of these, many helminthosporoid taxa known as parasites on graminicolous plants, or saprobes on woody substrates, were excluded due to their atypical features in *Helminthosporium* [18,22,23]. Nowadays, only 72 species have been accepted followed Siboe et al.’s [22] treatment [23,24,25,26,27,28,29,30], and one species, *H. solani* is an economically important pathogen that attacks the periderm of the potato tuber causing silver scurf disease [31]. Currently, genomic resources are limited in *Helminthosporium*, with only 42 species represented by DNA sequences in GenBank.

The southern region of China boasts complex geography, warm and humid climates, and abundant plant resources, which led to an extensive accumulation of various microbial species in the forest ecosystems. During our continuing surveys of saprobic fungi associated with plant debris in southern China, over 270 strains were isolated from dead branches, including several *Distoseptispora*-like and *Helminthosporium*-like fungi. Based on phylogenetic analyses combined with morphological comparisons, seven species of *Distoseptispora* and *Helminthosporium* were introduced, including six new species, namely *D. terrestris*, *D. wuyishanensis*, *D. zhejiangensis*, *H. ganzhouense*, *H. jiangxiense* and *H. saprophyticum*, as well as one known species, *H. velutinum*.

## 2. Materials and Methods

### 2.1. Sample Collection, Fungal Isolation and Morphological Observation

Dead branches were obtained from the subtropical forest ecosystems in southern China. The samples were placed in Ziploc™ sealed bags and taken to the laboratory. Subsequent treatment of the samples were performed according to the methods in Ma et al. [32]. Microscopic characters of colonies on samples surface was observed using a Motic SMZ-168 stereomicroscope (Motic China Group Co., Ltd., Xiamen, China), and then transferred onto a slide with lactic acid–phenol solutions (lactic acid, phenol, glycerin, sterile water in a ratio of 1:1:2:1) using sterile needles. The microscopic morphological characteristics was examined and recorded by the slide under an Olympus BX 53 light microscope equipped with an Olympus DP 27 digital camera (Olympus Optical Co., Ltd., Tokyo, Japan). A toothpick dipped in sterile water was used to collect conidia from the target colony on the surface of the dead branches under 5 × magnification. The culture and purification of the conidia were performed according to the method described by Liu et al. [14]. The morphological characteristics of the cultures (n = 3) were observed and documented after four weeks of incubation, including mycelial growth patterns, shape, size, and color. The voucher specimens and cultures were kept in the Herbarium of Jiangxi Agricultural University, Plant Pathology, Nanchang, China (HJAUP).

### 2.2. DNA Extraction, PCR Amplification and Sequencing

Genomic DNA of studied strains was extracted using a fungal genomic DNA extraction kit (Beijing Solarbio Science & Technology Co., Ltd., Beijing, China) following the method provided by instructions for use. Polymerase chain reaction (PCR) was performed by Genomic DNA of five loci including the internal transcribed spacer (ITS: ITS5/ITS4) [33], the large subunit ribosomal DNA (LSU: 28S1–F/28S3–R) [8], the small subunit ribosomal RNA (SSU: 18S–F/18S–R) [8], the partial second largest subunit of RNA polymerase II (*RPB2*: *RPB2*–5F2 [34] /fRPB2–7cR) [35], and the partial translation elongation factor 1-alpha (*TEF1:* EF1–983F/EF1–2218R) [36,37]. The PCR mixture (25 µL total volume) consisted of 9.5 µL of double-distilled water (ddH_2_O), 12.5 µL of 2× Power Taq PCR MasterMix, 1 µL per primer (forward and reverse), and 1 µL of DNA template. The PCR thermal cycling conditions are shown in Table 1. The quality of PCR products were verified on 1% agarose gel electrophoresis stained with ethidium bromide. PCR products were outsourced to Beijing Tsingke Biotechnology Co., Ltd., Beijing, China, for purification and DNA sequencing.

### 2.3. Phylogenetic Analyses

The newly obtained sequences were aligned with related sequences downloaded from GenBank (Table 2 includes ITS, LSU, *RPB2*, and *TEF1*; Table 3 includes ITS, LSU, *RPB2*, SSU, and *TEF1*) using the online program MAFFTv.7 [38] (https://mafft.cbrc.jp/alignment/server/index.html, accessed on 10 March 2025), followed by manual optimization to enhance the alignment quality and sequence approximation. Each single-locus phylogenetic analyses was performed, after which the aligned datasets of Table 2 (ITS, LSU, *RPB2*, and *TEF1*) and the aligned datasets of Table 3 (ITS, LSU, *RPB2*, SSU, and *TEF1*) were concatenated, respectively, using Phylosuite software v1.2.2 [39]. Based on the concatenated ITS–LSU–*RPB2*–*TEF1* sequence data as well as ITS–LSU–*RPB2*–SSU–*TEF1* sequence data, the multigene phylogenetic trees were constructed using Phylosuite software v1.2.2 [39]. Phylogenetic analyses were conducted using both maximum likelihood (ML) and Bayesian inference (BI) on the concatenated aligned datasets. The ModelFinder function was used to select the best fitting partition model [40], using the BIC criterion for constructing IQ-TREE and the AlCc criterion for MrBayes. IQ-TREE was used to infer the ML phylogenies [41] by a best partition model with 10,000 ultrafast bootstraps [42]. MrBayes 3.2.6 [43] was used to infer the BI phylogenies by a best partition model (two parallel runs, 2,000,000 generations), discarding the initial 25% of sampled data as burn-in. For the genus *Distoseptispora*, the likelihood scores were compared to select the final tree from suboptimal trees of each run in ML phylogenies, with the SYM + I + G4 model used for ITS, TIM2 + F + R4 for LSU and *TEF1*, and TIM3 + F + R3 for *RPB2*. The best-fit model of BI phylogenies was SYM + I + G4 for ITS, GTR + F + I + G4 for LSU, *RPB2* and *TEF1*. For the genus *Helminthosporium*, the best model of ML phylogenies was the TIM2e + I + G4 model used for ITS and LSU, TIM2 + F + I + G4 for *RPB2*, K2P + R2 for SSU, and TN + F + I + G4 for *TEF1*. The best-fit model of BI phylogenies was SYM + I + G4 for ITS, GTR + F + I + G4 for LSU, *RPB2* and *TEF1*, and HKY + F + G4 for SSU. The final tree were visualized using FigTree v. 1.4.4. And graphical refinement were using Adobe Illustrator CS v. 5. The newly obtained sequences in this study were submitted to GenBank.

## 3. Results

### 3.1. Molecular Phylogeny

**Figure 1:**
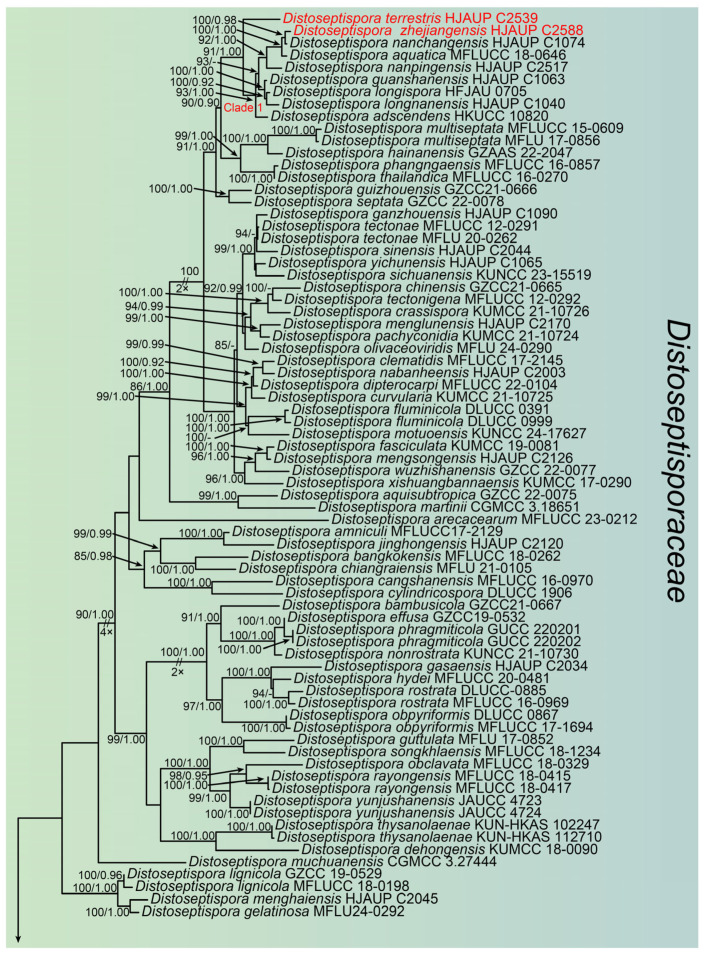

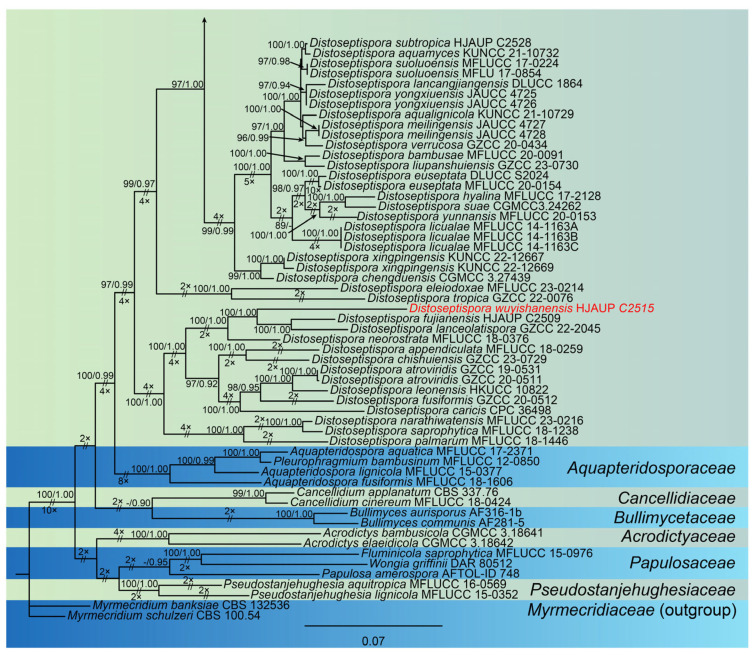
The phylogenetic tree of *Distoseptispora* and related families based on the combined ITS, LSU, *RPB2* and *TEF1* sequences. *Myrmecridium schulzeri* (CBS 100.54) and *M. banksiae* (CBS 132536) was outgroup. The ML bootstrap support values (>75%) and BI posterior probabilities (>0.90) are shown in the first and second positions, respectively. New species are shown in red. Some branches were shortened according to the indicated multipliers, and these are indicated by the symbol (//).

Based on the combined sequences of ITS, LSU, *RPB2* and *TEF1*, the phylogenetic tree was constructed to analyze the phylogenetic relationships of the three strains in this study. The family *Distoseptisporaceae* and related families (*Acrodictyaceae*, *Aquapteridosporaceae*, *Bullimycetaceae*, *Cancellidiaceae*, *Papulosaceae*, and *Pseudostanjehughesiaceae*) was used a total of 131 strains representing 109 species (Table 2). *Myrmecridium banksiae* (CBS 132536) and *M. schulzeri* (CBS 100.54) were used as the outgroup. The combined sequence consisted of 2759 nucleotide positions (ITS:1–581, LSU:582–1095, *RPB2*:1096–1872, and *TEF1*:1873–2759), comprising 1115 parsimony-informative sites, 192 singleton sites, 1452 constant sites, and 1466 distinct patterns. The phylogenetic reconstruction obtained from ML and BI analyses of the combined sequences showed essentially congruent topological structures. The best-scoring ML tree (lnL = −37642.042) was shown in Figure 1, with node support values indicated above each branch. The first and second values at each node represent ultrafast bootstrap support values from ML analysis and posterior probabilities from MrBayes analysis, respectively. Based on multi-locus phylogenetic analyses combined with morphological characteristics, the three strains in this study were classified into three novel species, namely *D. terrestris*, *D. wuyishanensis*, and *D. zhejiangensis*.

**Figure 2:**
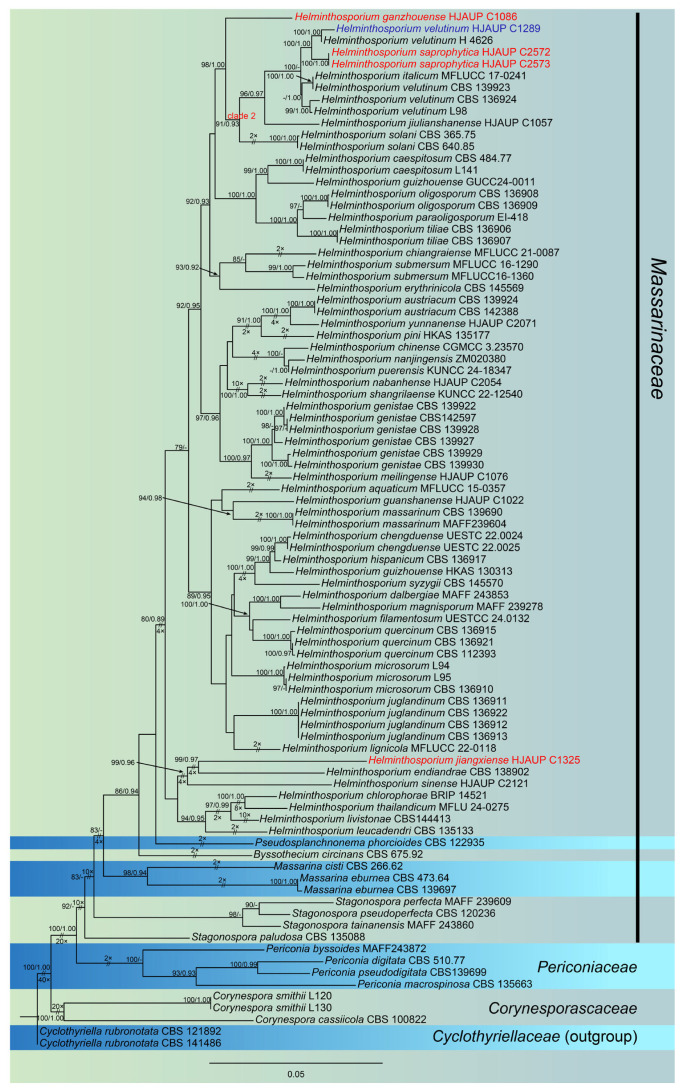
The phylogenetic tree of *Helminthosporium* and related families based on the combined ITS, LSU, *RPB2*, SSU and *TEF1* sequences. *Cyclothyriella rubronotata* (CBS 121892) and *C. rubronotata* (CBS 141486) was outgroup. The ML bootstrap support values (>75%) and BI posterior probabilities (>0.90) are shown in the first and second positions, respectively. New strains identified in this study are shown in blue; new species are shown in red. Some branches were shortened according to the indicated multipliers, and these are indicated by the symbol (//).

Based on the combined sequences of ITS, LSU, *RPB2*, SSU and *TEF1*, the phylogenetic tree was constructed to analyze the phylogenetic relationships of the five strains in this study. The family *Massarinaceae* and related families (*Periconiaceae*, *Corynesporascaceae* and *Cyclothyriellaceae*) was used a total of 86 strains representing 55 species (Table 3). *Cyclothyriellaceae rubronotata* (CBS 121892) and *C. rubronotata* (141486) were used as the outgroup. The combined sequence consisted of 3744 nucleotide positions (ITS:1–532, LSU:533–1054, *RPB2*:1055–2087, SSU:2088–2861, and *TEF1*:2862–3730), comprising 1058 parsimony-informative sites, 201 singleton sites, 2485 constant sites, and 1435 distinct patterns. The phylogenetic reconstruction obtained from ML and BI analyses of the combined sequences showed essentially similar topological structures. The best-scoring ML tree (lnL = −25759.369) was shown in Figure 2, with node support values indicated above each branch. The first and second values at each node represent ultrafast bootstrap support values from ML analysis and posterior probabilities from MrBayes analysis, respectively. Based on multi-locus phylogenetic analyses combined with morphological characteristics, the five strains in this study were classified into three new species, namely *H. ganzhouense*, *H. jiangxiense*, and *H. saprophyticum* and one known species, *H. velutinum*.

### 3.2. Taxonomy

**Figure 3:**
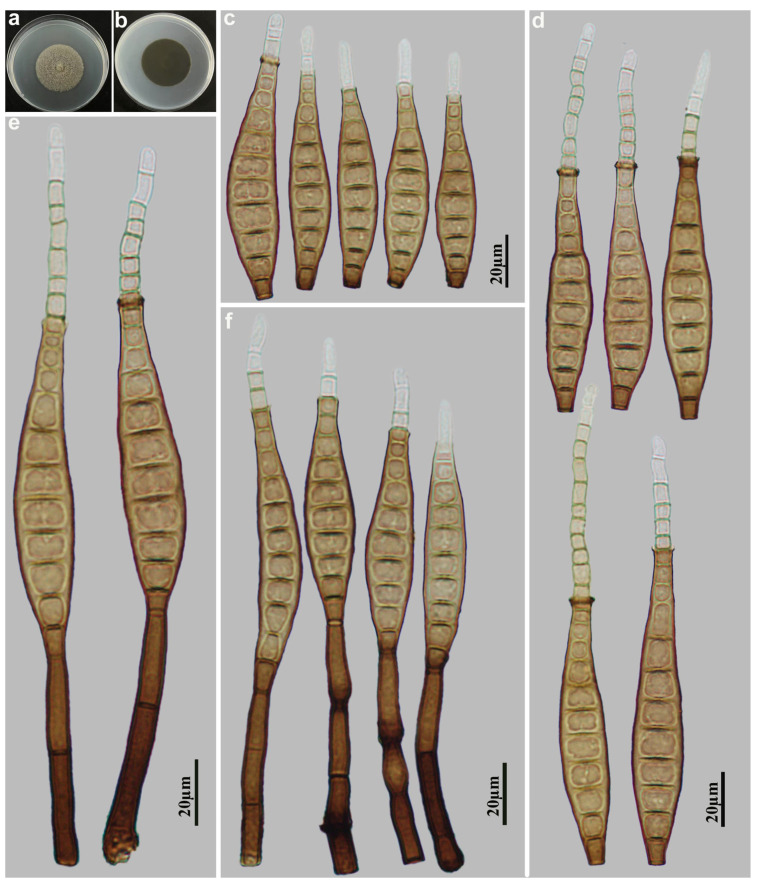
*Distoseptispora terrestris* (HJAUP M2539, holotype): (**a**) Colony on PDA after 4 weeks (from above); (**b**) Colony on PDA after 4 weeks (from below); (**c**,**d**) Conidia; (**e**,**f**) Conidiophores, conidiogenous cells and conidia.

*Distoseptispora terrestris* M.G. Liao & Jian Ma, sp. nov., Figure 3.

Index Fungorum number: IF903855

Etymology: The name refers to the fact that this fungus was isolated from a terrestrial habitat.

Holotype: HJAUP M2539

Description: Asexual morph on natural substrate. Colonies effuse, scattered, smooth, hairy, dark brown or black. Mycelium immersed and superficial, composed of septate, branched, pale brown hyphae. Conidiophores macronematous, mononematous, solitary or in groups of 2–3, unbranched, stralight or flexuous, smooth, 2–3-septate, cylindrical, brown to dark brown, paler towards the apex, determinate or sometimes with cylindrical, enteroblastic percurrent extensions, 52.5–128 × 6.3–8.8 µm ($\bar{x}$ = 76 × 7.3 µm, n = 15). Conidiogenous cells monoblastic, terminal, integrated, smooth, cylindrical, brown, with a flat apex. Conidial secession schizolytic. Conidia acrogenous, solitary, obclavate, straight or slightly curved, smooth, brown, 9–11-distoseptate, with a subhyaline to pale brown, euseptate rostrate regenerated from the terminal fracture of conidia, 54.5–91 × 11–18 µm ( $\bar{x}$ = 71.8 × 14 µm, n = 30), 8.8–10 µm in width at the truncate base, gradually tapering to 5–7.5 µm towards the apex. Sexual morph are unknown.

Culture characteristics: Colonies grown on PDA reaching 44 mm diameter from 28 to 42 days at 25 °C in dark conditions, circular, flat, surface grayish brown, with dense mycelium, and entire margin; reverse dark brown to black.

Material examined: China, Fujian Province, Nanping City, Wuyishan National Nature Reserve, 27°43′ N, 117°41′ E, on dead branches of unidentified plants under broad-leaved forest, 16 October 2023, Y.F. Hu (HJAUP M2539, holotype; HJAUP C2539, ex-type living culture).

Notes: Phylogenetic analyses shows that *D. terrestris* (HJAUP C2539) formed an independent lineage basal to Clade 1 with 91%ML/1.00BI bootstrap support. However, *D. terrestris* can be distinguished from the morphologically most similar species, *D. longnanensis* Y.F. Hu & Jian Ma [44] by its 9–11-distoseptate, wider conidia (11–18 µm vs. 8.2–14 µm wide) with euseptate rostrate regenerated from its terminal fracture, and further from *D. longnanensis* by 66 nucleotide differences (34/613 in ITS including 9 gaps, 7/555 in LSU including one gap, and 25/943 in *TEF1* including 17 gaps). In addition, *D. terrestris* also differs from other taxa in Clade 1 in the shape and size of conidia and conidiophores.

**Figure 4:**
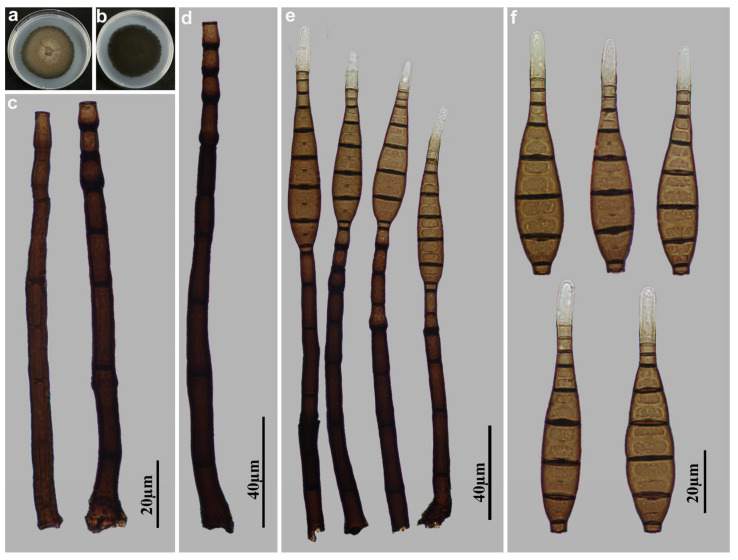
*Distoseptispora wuyishanensis* (HJAUP M2515, holotype): (**a**) Colony on PDA after 4 weeks (from above); (**b**) Colony on PDA after 4 weeks (from below); (**c**,**d**) Conidiophores; (**e**) Conidiophores, conidiogenous cells and conidia; (**f**) Conidia.

*Distoseptispora wuyishanensis* M.G. Liao & Jian Ma, sp. nov., Figure 4.

Index Fungorum number: IF903856

Etymology: The name refers to Wuyishan National Nature Reserve, the locality where the fungus was collected.

Holotype: HJAUP M2515

Description: Asexual morph on natural substrate. Colonies effuse, scattered, smooth, hairy, dark brown or black. Mycelium immersed and superficial, composed of septate, branched, pale brown hyphae. Conidiophores macronematous, mononematous, solitary, unbranched, cylindrical, stralight or flexuous, 5–11-septate, brown to dark brown, paler towards the apex, determinate or with several cylindrical, enteroblastic percurrent extensions, 85.5–192 × 5.5–9.1 µm ($\bar{x}$ = 130.9 × 7.3 µm, n = 15). Conidiogenous cells monoblastic, terminal, integrated, cylindrical, smooth, brown, with a flat apex. Conidial secession schizolytic. Conidia acrogenous, solitary, obclavate, fusiform, straight or slightly curved, brown, 5–8-euseptate and 5-distoseptate, smooth, rostrate, 54.5–91 × 11–18 µm ($\bar{x}$ = 71.8 × 14 µm, n = 30), 4.2–5.5 µm in width at the truncate base, gradually tapering to 3.3–4.5 µm towards the apex. Sexual morph are unknown.

Culture characteristics: Colonies grown on PDA reaching 57 mm diameter from 28 to 42 days at 25 °C in dark conditions, circular, flat, surface yellowish brown, with dense mycelium, dark brown at the margin; reverse black with sparse edges.

Material examined: China, Fujian Province, Nanping City, Wuyishan National Nature Reserve, 27°43′ N, 117°41′ E, on dead branches of unidentified plants under broad-leaved forest, 16 October 2023, Y.F. Hu (HJAUP M2515, holotype; HJAUP C2515, ex-type living culture).

Notes: Phylogenetic analyses shows that *D. wuyishanensis* (HJAUP C2515) clusters as an independent clade sister to *D. fujianensis* (HJAUP C2509) and *D*. *lanceolatispora* (GZCC 22–2045) with 100%ML/1.00BI bootstrap support. The BLASTn shows that the nucleotide comparison of *D. wuyishanensis* (HJAUP C2515) and *D. fujianensis* (HJAUP C2509) in 256 nucleotide differences (55/608 in ITS including 16 gaps, 39/582 in LSU including 4 gaps, 116/946 in *RPB2* including one gap, 46/922 in *TEF1* including one gap). The sequences comparison of *D. lanceolatispora* (GZCC 22–2045) share 237 nucleotide differences (53/528 in ITS including 20 gaps, 40/572 in LSU including 3 gaps, 107/896 in *RPB2* including one gap, 37/906 in *TEF1* including one gap). Moreover, *D. wuyishanensis* differs from *D. fujianensis* M.G. Liao & Jian Ma [45] and *D*. *lanceolatispora* X.M. Chen & Y.Z. Lu [46] by its conidia with both eusepta and distosepta, and further from *D*. *fujianensis* by its bigger conidiophores (85.5–192 × 5.5–9.1 µm vs. 86–127 × 4.5–6 μm) and conidia (54.5–91 × 11–18 µm vs. 28–47 × 8–14 μm), and from *D*. *lanceolatispora* by its fusiform, smooth, wider conidia (11–18 µm vs. 9.5–15 μm).

**Figure 5:**
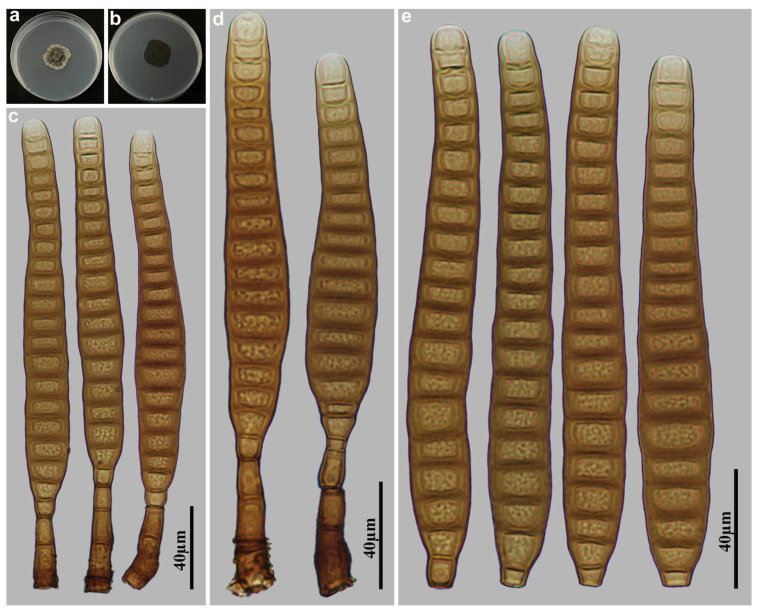
*Distoseptispora zhejiangensis* (HJAUP M2588, holotype): (**a**) Colony on PDA after 4 weeks (from above); (**b**) Colony on PDA after 4 weeks (from below); (**c**,**d**) Conidiophores, conidiogenous cells and conidiaconidia; (**e**) Conidia.

*Distoseptispora zhejiangensis* M.G. Liao & Jian Ma, sp. nov., Figure 5.

MycoBank number: MB859236

Etymology: The name refers to Zhejiang, the province where the fungus was collected.

Holotype: HJAUP M2588

Description: Asexual morph on natural substrate. Colonies effuse, scattered, smooth, hairy, dark brown or black. Mycelium immersed and superficial, composed of septate, branched, pale brown hyphae. Conidiophores macronematous, mononematous, solitary, unbranched, smooth, cylindrical, stralight, 1–3-septate, brown, 16–56 × 13.5–20 µm ($\bar{x}$ = 38 × 9.7 µm, n = 15). Conidiogenous cells monoblastic, terminal, integrated, cylindrical, smooth, brown, with a flat apex. Conidial secession schizolytic. Conidia acrogenous, solitary, obclavate, straight or slightly curved, 13–20-distoseptate, smooth, brown, paler towards the apex, 128.1–261.7 × 18.7–26.7 µm ($\bar{x}$ = 161.1 × 23.5 µm, n = 30), 5.7–8.6 µm in width at the truncate base, gradually tapering to 7.1–14 µm towards the apex. Sexual morph are unknown.

Culture characteristics: Colonies grown on PDA reaching 27 mm diameter from 28 to 42 days at 25 °C in dark conditions, irregular circular, flat, surface velvety, with abundant aerial mycelium, dark olivaceous, paler towards the margin; reverse black.

Material examined: China, Zhejiang Province, Quzhou City: Meishudi Scenic, 29°08′ N, 118°69′ E, on dead branches of unidentified plants under broad-leaved forest, 21 June 2022, Y.F. Hu (HJAUP M2588, holotype; HJAUP C2588, ex-type living culture).

Notes: Phylogenetic analyses shows that *D. zhejiangensis* (HJAUP C2588) clusters with *D. nanchangensis* (HJAUP C1074) with 100%ML/0.98BI bootstrap support. The BLASTn shows that the nucleotide comparison of *D. zhejiangensis* (HJAUP C2588) and *D. nanchangensis* (HJAUP C1074) in 6 nucleotide differences (5/588 in ITS including one gap, 1/932 in *TEF1*). Moreover, *D. zhejiangensis* differs from *D. nanchangensis* Y.F. Hu & Jian Ma [44] in its shorter and wider conidiophores (16–56 × 13.5–20 µm vs. 18.2–76.4 × 5.5–8 µm) and shorter and wider conidia (128.1–261.7 × 18.7–26.7 µm vs. 149.1–292.7 × 10.9–17.8 µm) with fewer distosepta (13–20 vs. 17–43). In addition, the conidia of *D. nanchangensis* sometimes bear percurrent regeneration forming a secondary conidium, or a germ tube or short germination hypha from the conidial apex.

**Figure 6:**
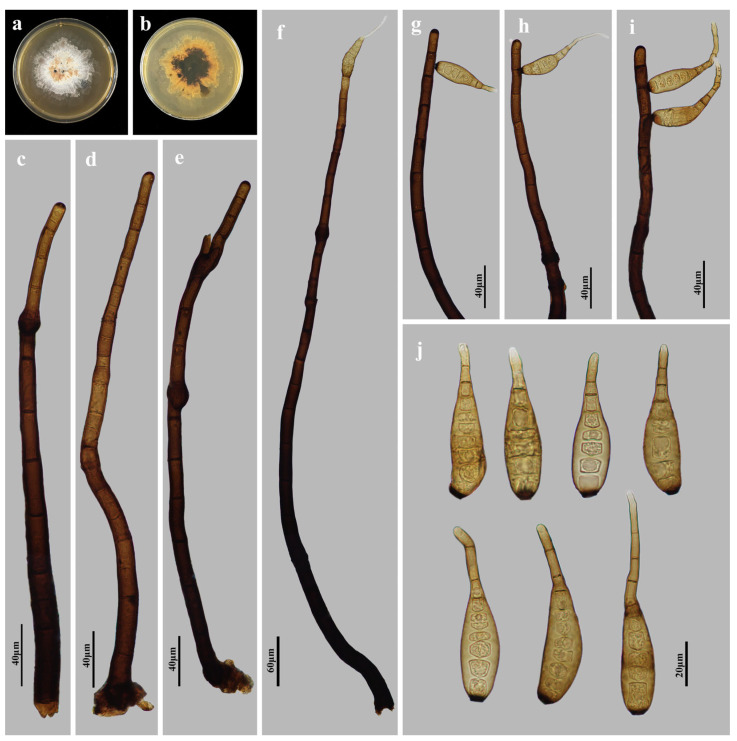
*Helminthosporium ganzhouense* (HJAUP M1086, holotype): (**a**) Colony on PDA after 4 weeks (from above); (**b**) Colony on PDA after 4 weeks (from below); (**c**–**e**) Conidiophores; (**f**–**i**) Conidiophores, conidiogenous cells and conidia; (**j**) Conidia.

*Helminthosporium ganzhouense* M.G. Liao & Jian Ma, sp. nov., Figure 6.

MycoBank number: MB859237

Etymology: The name refers to Ganzhou city, the locality where the fungus was collected.

Holotype: HJAUP M1086

Description: Asexual morph on natural substrate. Colonies effuse, scattered, smooth, hairy, dark brown or black. Mycelium immersed and superficial, composed of septate, branched, pale brown hyphae. Conidiophores macronematous, mononematous, solitary, unbranched, erect, curved, smooth, cylindrical, dark brown, paler towards the apex, 10–24-septate, enteroblastic percurrent extensions, with distinct pores in subapical region, 312–428–(744) × 10.5–20 µm ($\bar{x}$ = 420 × 14.4 µm, n = 15). Conidiogenous cells polytretic, terminal, cylindrical, integrated, brown. Conidial secession schizolytic. Conidia acropleurogenous, solitary, rostrate, obclavate, straight or curved, pale brown, 8–13-distoseptate, 62–96 × 12–16 µm ($\bar{x}$ = 78 × 14.4 µm, n = 30), 4–5.9 µm in width at the truncate base, gradually tapering to 1.2–4 µm towards the apex. Sexual morph are unknown.

Culture characteristics: Colonies grown on PDA reaching 54 mm diameter from 28 to 42 days at 25 °C in dark conditions, irregular circular, surface gray-white with yellow-brown in the center; reverse rosy-brown with dark brown center.

Material examined: China, Jiangxi Province, Ganzhou City: Jiulianshan National Nature Reserve, 24°31′ N, 114°27′ E, on dead branches of unidentified plants under broad-leaved forest, 29 June 2022, Y.F. Hu (HJAUP M1086, holotype; HJAUP C1086, ex-type living culture).

Notes: Phylogenetic analyses shows that *H. ganzhouense* (HJAUP C1086) belongs to *Helminthosporium* and forms a distinct lineage sister to Clade 2 with 98%ML/1.00BI bootstrap support. However, *H. ganzhouense* differs from the morphologically most similar species, *H. solani* Durieu & Mont. [19,47] in its shorter and wider conidiophores (312–744 × 10.5–20 vs. 120–600 × 9–15 µm) and bigger conidia (62–96 × 12–16 µm vs. 24–85 × 7–11 µm) with more distosepta (8–13 vs. 2–8), and further from *H. solani* (CBS 365.75) by 93 nucleotide differences (27/568 in ITS including 3 gaps, 9/558 in LSU including 2 gaps, and 57/985 in *TEF1*). In addition, *H. ganzhouense* also differs from other taxa in Clade 2 in the size of conidiophores and conidia.

**Figure 7:**
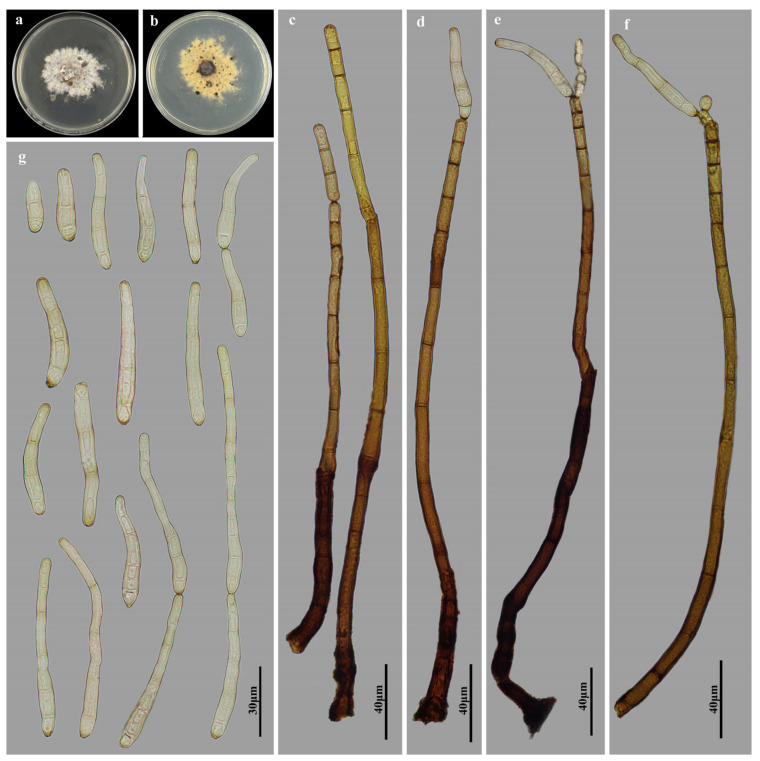
*Helminthosporium jiangxiense* (HJAUP M1325, holotype): (**a**) Colony on PDA after 4 weeks (from above); (**b**) Colony on PDA after 4 weeks (from below); (**c**) Conidiophores and conidiogenous cells; (**d**–**f**) Conidiophores, conidiogenous cells and conidia; (**g**) Conidia.

*Helminthosporium jiangxiense* M.G. Liao & Jian Ma, sp. nov., Figure 7.

MycoBank number: MB859238

Etymology: The name refers to Jiangxi, the province where the fungus was collected.

Holotype: HJAUP M1325

Description: Asexual morph on natural substrate. Colonies effuse, scattered, smooth, hairy, dark brown or black. Mycelium immersed and superficial, composed of septate, branched, pale brown hyphae. Conidiophores macronematous, mononematous, solitary, unbranched, erect, cylindrical, flexuous, 10–16-septate, dark brown to brown, paler towards the apex, enteroblastic percurrent extensions, with distinct pores in subapical region, 222.5–350 × 6.3–8 µm ($\bar{x}$ = 278.1 × 7.3 µm, n = 10). Conidiogenous cells polytretic, terminal and intercalary, integrated, cylindrical, brown. Conidial secession schizolytic. Conidia solitary or in short chains, acropleurogenous, obclavate, straight or curved, pale brown, smooth, 2–10-distoseptate, 30–132.5 × 5–9.5 µm ($\bar{x}$ = 55 × 6.5 µm, n = 30), 1.3–2.5 µm in width at the truncate base, gradually tapering to 2.5–3.8 µm towards the apex. Sexual morph are unknown.

Culture characteristics: Colonies grown on PDA reaching 54 mm diameter from 28 to 42 days at 25 °C in dark conditions, irregular circular, surface gray-white with dark brown; reverse rosy-brown with dark brown center.

Material examined: China, Jiangxi Province, Ganzhou City: Jiulianshan National Nature Reserve, 24°31′ N, 114°27′ E, on dead branches of unidentified plants under broad-leaved forest, 29 June 2022, Y.F. Hu (HJAUP M1325, holotype; HJAUP C1325, ex-type living culture).

Notes: Phylogenetic analyses shows that *H. jiangxiense* (HJAUP C1325) clusters with *H. endiandrae* (CBS 138902) with 99%ML/0.97BI bootstrap support. The BLASTn shows that the nucleotide comparison of *H. jiangxiense* (HJAUP C1325) and *H. endiandrae* (CBS 138902) show 93 nucleotide differences (73/593 in ITS including 30 gaps, 20/575 in LSU including 2 gaps). Moreover, *H. jiangxiense* differs from *H. endiandrae* (Crous & Summerell) Voglmayr & Jaklitsch [18] in its polytretic conidiogenous cell, bigger conidiophores (222.5–350 × 6.3–8 µm vs. 200–300 × 5–7 µm) and longer conidia (30–132.5 µm vs. 35–57 µm) with more distosepta (2–10 vs. 3–4).

**Figure 8:**
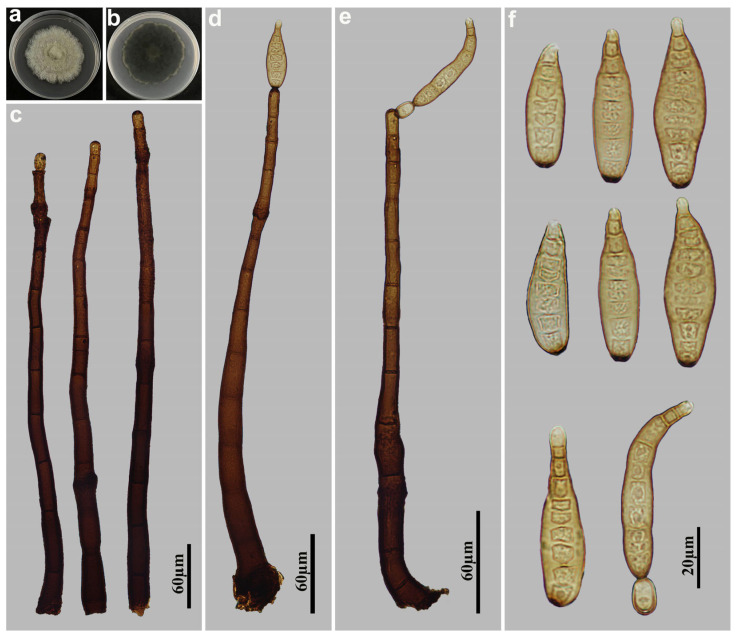
*Helminthosporium saprophyticum* (HJAUP M2572, holotype): (**a**) Colony on PDA after 4 weeks (from above); (**b**) Colony on PDA after 4 weeks (from below); (**c**) Conidiophores; (**d**,**e**) Conidiophores, conidiogenous cells and conidia; (**f**) Conidia.

*Helminthosporium saprophyticum* M.G. Liao & Jian Ma, sp. nov., Figure 8.

MycoBank number: MB859239

Etymology: The epithet refers to the saprophytic habit on dead branches.

Holotype: HJAUP M2572

Description: Asexual morph on natural substrate. Colonies effuse, scattered, smooth, hairy, dark brown or black. Mycelium immersed and superficial, composed of septate, branched, pale brown hyphae. Conidiophores macronematous, mononematous, solitary, unbranched, cylindrical, erect, flexuous, dark brown, paler towards the apex, enteroblastic percurrent extensions, with distinct pores in subapical region, 10–16 septate, 285.7–542 × 14.3–20 µm ($\bar{x}$ = 417.1 × 18.8 µm, n = 15). Conidiogenous cells polytretic, terminal and intercalary, integrated, cylindrical, brown. Conidial secession schizolytic. Conidia acropleurogenous, solitary, rostrate, obclavate, straight or curved, pale brown, 6–10-distoseptate, 37.1–57.1 × 10–17.1 µm ($\bar{x}$ = 47.7 × 13.1 µm, n = 30), 3.2–5.8 µm in width at the truncate base, gradually tapering to 2.9–3.4 µm towards the apex. Sexual morph are unknown.

Culture characteristics: Colonies grown on PDA reaching 65 mm diameter from 28 to 42 days in the incubation at 25 °C and dark conditions, subcircular, surface velvety, with abundant aerial mycelium, grayish brown, pale at the margin; reverse black with pale margin.

Material examined: China, Jiangxi Province, Ganzhou City: Jiulianshan National Nature Reserve, 24°31′ N, 114°27′ E, on dead branches of unidentified plants under broad-leaved forest, 29 June 2022, Y.F. Hu (HJAUP M2572, holotype; HJAUP C2572 = HJAUP C2573, ex-type living culture).

Notes: Phylogenetic analyses shows that *H. saprophyticum* (HJAUP C2572) and *H. saprophyticum* (HJAUP C2573) clusters with *H. velutinum* (H 4626 and HJAUP C1289) with 100%ML/1.00BI bootstrap support. The BLASTn shows that the nucleotide comparison of *H. saprophyticum* (HJAUP C2572) and *H. velutinum* (H 4626) in 11 nucleotide differences (2/524 in ITS, 1/579 in LSU, 3/472 in SSU including one gap, 5/923 in *TEF1*). Moreover, *H. saprophyticum* differs from *H. velutinum* Link [17,18] in having smaller conidiophores (285.7–542 × 14.3–20 µm vs. 340–698 × 14–26 µm), and smaller conidia (37.1–57.1 × 10–17.1 µm vs. 56–89 ×14.3–18.5 µm) with fewer distosepta (6–10 vs. 6–18).

*Helminthosporium velutinum* Link [as ‘*Helmisporium*’], Mag. Gesell. naturf. Freunde, Berlin 3(1–2): 10, (1809), Figure 9.

Description: Asexual morph on natural substrate. Colonies effuse, scattered, smooth, hairy, dark brown or black. Mycelium immersed and superficial, composed of septate, branched, pale brown hyphae. Conidiophores macronematous, mononematous, solitary, erect, unbranched, cylindrical, flexuous, dark brown, paler towards the apex, 13–20-septate, enteroblastic percurrent extensions, with distinct pores in subapical region, 464–616 × 12–16 µm, ($\bar{x}$ = 552 × 14.8 µm, n = 10). Conidiogenous cells polytretic, terminal and intercalary, integrated, cylindrical, brown. Conidial secession schizolytic. Conidia acropleurogenous, solitary, rostrate, obclavate, straight or curved, smooth, pale brown, 4–13-distoseptate, constricted at the distoseptate, especially at the apex, 40–96 × 8–16 µm ($\bar{x}$ = 68 × 12 µm, n = 30), 4.2–5.9 µm in width at the truncate base, gradually tapering to 2.8–5.2 µm toward the apex. Sexual morph are unknown.

Culture characteristics: Colonies grown on PDA reaching 81 mm diameter from 28 to 42 days at 25 °C in dark conditions, circular, surface creamish white with black margins, velvety mycelium in center; reverse black with sparse edges.

Material examined: China, Jiangxi Province, Ganzhou City: Jiulianshan National Nature Reserve, 24°31′ N, 114°27′ E, on dead branches of unidentified plants under broad-leaved forest, 29 June 2022, Y.F. Hu, HJAUP M1289; HJAUP C1289 (living culture).

Notes: *Helminthosporium velutinum*, the generic type, is a cosmopolitan species, and has been recorded from a wide range of woody and herbaceous plants [18]. Phylogenetic analyses shows that our new isolate (HJAUP C1289) clusters with *H. velutinum* (H 4626) with 100%ML/1.00BI bootstrap support. Morphologically, our isolate fits well with the description of *H. velutinum* by Voglmayr & Jaklitsch [18], except for the narrower conidiophores (12–16 µm vs. 14–26 µm). Comparative analysis of their nucleotide sequences revealed no genetic differences in the ITS (524/524, no gaps), LSU (552/552, no gaps), SSU (421/421, no gaps), and *TEF1* (923/923, no gaps) regions. Based on the high morphological similarity and no nucleotide differences, we identified our isolate (HJAUP M1289) as *H. velutinum*.

## 4. Discussion

In recent years, studies on microfungi have received extensive attention, and numerous published information about their taxonomy are recorded in China [48,49,50,51,52]. During the ongoing study, we collected saprophytic microfungi from dead branches in southern China, and highly phylogenetic support and significant morphological comparison showed six novel species, namely *Distoseptispora terrestris*, *D. wuyishanensis*, *D. zhejiangensis*, *Helminthosporium ganzhouense*, *H. jiangxiense*, and *H. saprophyticum*, and one known species, *H. velutinum*.

*Distoseptispora* is one of the sporidesmium-like genera introduced by Su et al. [7] based on molecular phylogenetic analyses combined with morphology. *Distoseptispora* species were well described in terms of their taxonomic placements and genetic relationship by significant morphological characteristics and phylogenetic analyses. However, the research conducted on *Distoseptispora* has no universally accepted Standards for genetic barcode selection in phylogenetic analyses [14], which result in a lack of correlation between phylogenetic relationships and morphological analysis for some *Distoseptispora* species. For instance, *D. lanceolatispora* cluster with *D. neorostrata* in a subclade with highly genetic relationship, but significant morphological variations were observed, specifically in conidiophores, conidiogenous cells, and conidia [46,53]. The limited phylogenetic resolution at the genus level may be attributed to the exclusive use of ribosomal genes in the analysis of *D. neorostrata*. The holotype of *D. bambusae* (MFLU 20-0261) exhibited distinct morphological differences from phylogenetic strains (MFLU 17-1653 and HKAS 125826) in the characteristics of conidiophores, conidiogenous cells, and conidia [54,55]. Under the current phylogenetic framework, traditional taxonomic characteristics such as conidiogenesis may no longer play a decisive role in species delimitation within sporidesmium-like genera [45]. A comprehensive investigation incorporating ribosomal genes and additional genomic loci is required to clarify the relationship between phylogenetic affiliations and morphological traits.

The genus *Helminthosporium* was introduced by Link based on morphological characters. To date, more than 780 epithets for *Helminthosporium* are listed in Index Fungorum [15], and only 45 species, including our three new species, were provided with DNA sequences. Most *Helminthosporium* species were reported before sequencing technology, resulting in a lack of molecular data. It is questionable to identify *Helminthosporium* species and related genera only by reference to morphological characteristics, host, and environment. Voglmayr & Jaklitsch [18] investigate the phylogenetic relationships of *Helminthosporium* and its related genera, *Corynespora* and *Exosporium* based on phylogenetic analyses of combined SSU, ITS, LSU, *RPB2* and *TEF1* sequence data, synonymised *Exosporium* with *Helminthosporium*, and transformed four *Corynespora* species to *Helminthosporium*. Since then, the number of *Helminthosporium* species is steadily increasing, and all described species were introduced using multilocus phylogenetic analyses. However, recent studies indicated that the phylogenetic analysis for *Helminthosporium* species have no universally accepted standards in selecting barcodes [56] and based on only ribosomal genes may be insufficient in resolving the phylogeny of *Helminthosporium* and related genera within Massarinaceae, as together with *RPB2* and/ or *TEF1* showed more powerful resolution in species delineation and higher bootstrap support values for most clades [18,23,27,29]. Considering this phenomenon, we conducted phylogenetic analyses using ITS, LSU, *RPB2*, SSU and *TEF1*, and proposed three new *Helminthosporium* species, and one known species, *H. velutinum* in this study.

Current studies on *Distoseptispora* and *Helminthosporium* have predominantly focused on the taxonomy of their asexual morphs, with most specimens collected from submerged wood, dead twigs and plant leaves. However, few studies have investigated their ecological functions in relation to the specific substrates. Current knowledge regarding their functional roles in decomposition, nutrient cycling, geographical distribution patterns, ecological adaptability, host associations, substrate specificity, and teleomorph-anamorph relationships remains relatively limited. As a result, their precise contributions to ecosystem functioning cannot yet be quantitatively assessed [44]. Several *Helminthosporium* species have been isolated from plant leaves as endophytes or pathogens. By colonizing plant tissues, endophytic fungi support host development and increase resilience to environmental stresses [57,58], thereby improving the community structure of ecosystems. In contrast, *H. solani* as a pathogen mainly causes blemishes in the periderm of potato tubers [22], which has emerged as a significant economically important plant disease in 1990s [59,60]. Therefore, conducting surveys across diverse geographical regions, ecological environments, and vegetation types will contribute to the conservation of species diversity in *Distoseptispora* and *Helminthosporium*, while facilitating the clarification of their taxonomic status based on phylogenetic analyses combined with morphological characterization.

## Figures and Tables

**Figure 9:**
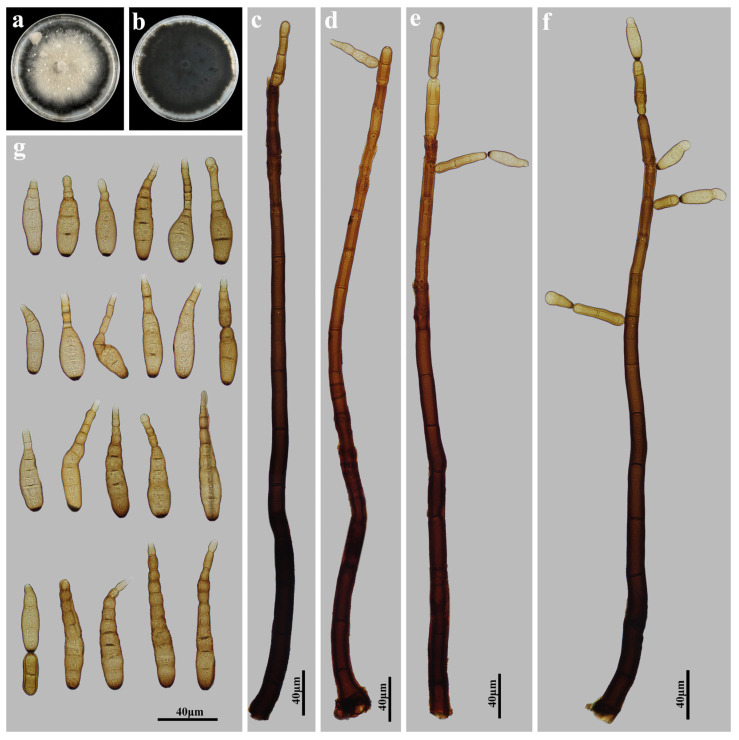
*Helminthosporium velutinum* (HJAUP M1289): (**a**) Colony on PDA after 4 weeks (from above); (**b**) Colony on PDA after 4 weeks (from below); (**c**) Conidiophore and conidiogenous cells; (**d**–**f**) Conidiophores, conidiogenous cells and conidia; (**g**) Conidia.

**Table 1:** The PCR thermal cycling conditions.

Locus	Initial Denaturation	Denaturation	Annealing	Elongation	Final Extension
ITS	94 °C/3 min	94 °C/15 s	54 °C/15 s	72 °C/30 s	72 °C/10 min
LSU	94 °C/3 min	94 °C/15 s	54 °C/15 s	72 °C/30 s	72 °C/10 min
*RPB2*	94 °C/3 min	94 °C/15 s	59 °C/50 s	72 °C/30 s	72 °C/10 min
SSU	94 °C/3 min	94 °C/15 s	54 °C/15 s	72 °C/30 s	72 °C/10 min
*TEF1*	94 °C/3 min	94 °C/15 s	54 °C/15 s	72 °C/30 s	72 °C/10 min

Note: Repeat steps of denaturation, annealing, elongation for 35 cycles.

**Table 2:** *Distoseptispora* species and relevant species with their corresponding GenBank accession numbers used in the phylogenetic analyses of this study. The ex-type strain are indicated using “^T^” after strain numbers; “—” stands for no sequence data in GenBank.

Taxon	Strain Number	GenBank Accession Numbers			
		ITS	LSU	*RPB2*	*TEF1*
*Acrodictys bambusicola*	CGMCC 3.18641 ^T^	KU999973	KX033564	—	—
*A. elaeidicola*	CGMCC 3.18642	KU999978	KX033569	—	—
*Aquapteridospora aquatica*	MFLUCC 17–2371 ^T^	MW286493	MW287767	—	—
*A. fusiformis*	MFLUCC 18–1606 ^T^	MK828652	MK849798	—	MN194056
*A. lignicola*	MFLUCC 15–0377 ^T^	MZ868774	KU221018	MZ892986	MZ892980
*Bullimyces aurisporus*	AF316–1b ^T^	—	JF775590	—	—
*B. communis*	AF281–5	—	JF775587	—	—
*Cancellidium applanatum*	CBS 337.76 ^T^	MH860985	MH872755	—	—
*Cancellidium cinereum*	MFLUCC 18–0424 ^T^	MT370353	MT370363	MT370486	MT370488
*Distoseptispora adscendens*	HKUCC 10820	—	DQ408561	DQ435092	—
*D. amniculi*	MFLUCC 17–2129 ^T^	MZ868770	MZ868761	MZ892982	—
*D. appendiculata*	MFLUCC 18–0259 ^T^	MN163009	MN163023	—	MN174866
*D. aqualignicola*	KUNCC 21–10729 ^T^	OK341186	ON400845	OP413474	OP413480
*D. aquamyces*	KUNCC 21–10732 ^T^	OK341187	OK341199	OP413476	OP413482
*D. aquatica*	MFLUCC 18–0646	MK828648	MK849793	—	MN194052
*D. aquisubtropica*	GZCC 22–0075 ^T^	ON527933	ON527941	ON533685	ON533677
*D. arecacearum*	MFLUCC 23–0212	OR354399	OR510860	OR481048	OR481045
*D. atroviridis*	GZCC 20–0511 ^T^	MZ868772	MZ868763	MZ892984	MZ892978
*D. atroviridis*	GZCC 19–0531	MW133915	MZ227223	—	MZ206155
*D. bambusae*	MFLUCC 20–0091 ^T^	MT232713	MT232718	MT232881	MT232880
*D. bambusicola*	GZCC 21–0667 ^T^	MZ474873	MZ474872	—	OM272845
*D. bangkokensis*	MFLUCC 18–0262 ^T^	MZ518205	MZ518206	—	OK067246
*D. cangshanensis*	MFLUCC 16–0970 ^T^	MG979754	MG979761	—	MG988419
*D. caricis*	CPC 36498 ^T^	MN562124	MN567632	MN556805	—
*D. chinensis*	GZCC 21–0665 ^T^	MZ474871	MZ474867	—	MZ501609
*D. clematidis*	MFLUCC 17–2145 ^T^	MT310661	MT214617	MT394721	—
*D. chengduensis*	CGMCC 3.27439 ^T^	PQ067828	PQ067744	—	PQ278565
*D. chiangraiensis*	MFLU 21-0105 ^T^	MZ890145	MZ890139	—	MZ892970
*D. chishuiensis*	GZCC 23-0729 ^T^	PP663310	PP584670	—	PP584767
*D. crassispora*	KUMCC 21–10726 ^T^	OK310698	OK341196	OP413473	OP413479
*D. curvularia*	KUMCC 21–10725 ^T^	OK310697	OK341195	OP413472	OP413478
*D. cylindricospora*	DLUCC 1906 ^T^	OK491122	OK513523	—	OK524220
*D. dehongensis*	KUMCC 18–0090 ^T^	MK085061	MK079662	—	MK087659
*D. dipterocarpi*	MFLUCC 22–0104 ^T^	OP600053	OP600052	OP595140	—
*D. effusa*	GZCC 19–0532 ^T^	MW133916	MZ227224	—	MZ206156
*D. eleiodoxae*	MFLUCC 23–0214	OR354398	OR510859	OR481047	OR481044
*D. euseptata*	MFLUCC 20–0154 ^T^	MW081539	MW081544	MW151860	—
*D. euseptata*	DLUCC S2024	MW081540	MW081545	MW084996	MW084994
*D. fasciculata*	KUMCC 19–0081 ^T^	MW286501	MW287775	—	MW396656
*D. fluminicola*	DLUCC 0391	MG979755	MG979762	—	MG988420
*D. fluminicola*	DLUCC 0999	MG979756	MG979763	—	MG988421
*D. fujianensis*	HJAUP C2509 ^T^	PQ211095	PQ211103	PQ303679	PQ303682
*D. fusiformis*	GZCC 20–0512 ^T^	MZ868773	MZ868764	MZ892985	MZ892979
*D. ganzhouensis*	HJAUP C1090 ^T^	PQ211100	PQ211108	—	PQ303687
*D. gasaensis*	HJAUP C2034 ^T^	OQ942896	OQ942891	—	OQ944455
*D. guanshanensis*	HJAUP C1063 ^T^	OQ942894	OQ942898	OQ944458	OQ944452
*D. guizhouensis*	GZCC 21–0666 ^T^	MZ474868	MZ474869	MZ501611	MZ501610
*D. guttulata*	MFLU 17–0852 ^T^	MF077543	MF077554	—	MF135651
*D. hainanensis*	GZCC 22-2047 ^T^	OR427328	OR438894	OR449119	OR449122
*D. hyalina*	MFLUCC 17–2128 ^T^	MZ868769	MZ868760	MZ892981	MZ892976
*D. hydei*	MFLUCC 20–0481 ^T^	MT734661	MT742830	MT767128	—
*D. jinghongensis*	HJAUP C2120 ^T^	OQ942897	OQ942893	—	OQ944456
*D. lancangjiangensis*	DLUCC 1864 ^T^	MW723055	MW879522	—	—
*D. lanceolatispora*	GZCC 22-2045 ^T^	OR427329	OR43BB95	OR449120	OR449123
*D. leonensis*	HKUCC 10822	—	DQ408566	DQ435089	—
*D. licualae*	MFLUCC 14–1163A ^T^	ON650686	ON650675	—	ON734007
*D. licualae*	MFLUCC 14–1163B ^T^	ON650687	ON650676	—	ON734008
*D. licualae*	MFLUCC 14–1163C ^T^	ON650688	ON650677	—	—
*D. lignicola*	MFLUCC 18–0198 ^T^	MK828651	MK849797	—	—
*D. lignicola*	GZCC 19–0529	MW133911	MZ227219	—	MZ206152
*D. liupanshuiensis*	GZCC 23-0730 ^T^	PP663309	PP584669	—	PP584766
*D. longispora*	HFJAU 0705 ^T^	MH555359	MH555357	—	—
*D. longnanensis*	HJAUP C1040 ^T^	OQ942887	OQ942886	—	OQ944451
*D. martinii*	CGMCC 3.18651 ^T^	KU999975	KX033566	—	—
*D. meilingensis*	JAUCC 4727 ^T^	OK562390	OK562396	—	OK562408
*D. meilingensis*	JAUCC 4728 ^T^	OK562391	OK562397	—	OK562409
*D. menghaiensis*	HJAUP C2045 ^T^	OQ942890	OQ942900	—	—
*D. menglunensis*	HJAUP C2170 ^T^	OQ942899	OQ942888	OQ944461	OQ944457
*D. mengsongensis*	HJAUP C2126 ^T^	OP787876	OP787874	—	OP961937
*D. motuoensis*	KUNCC24-17628 ^T^	PP600327	PP621731	—	PP639546
*D. muchuanensis*	CGMCC 3.27444 ^T^	PQ067834	PQ067750	—	PQ278571
*D. multiseptata*	MFLUCC 15–0609 ^T^	KX710145	KX710140	—	MF135659
*D. multiseptata*	MFLU 17–0856	MF077544	MF077555	MF135644	MF135652
*D. nabanheensis*	HJAUP C2003 ^T^	OP787873	OP787877	—	OP961935
*D. nanchangensis*	HJAUP C1074 ^T^	OQ942889	OQ942895	OQ944460	OQ944454
*D. nanpingensis*	HJAUP C2517 ^T^	PQ211096	PQ211104	PQ303678	—
*D. narathiwatensis*	MFLUCC 23–0216	OR354400	OR510861	OR481049	OR481046
*D. neorostrata*	MFLUCC 18–0376 ^T^	MN163008	MN163017	—	—
*D. nonrostrata*	KUNCC 21–10730 ^T^	OK310699	OK341198	OP413475	OP413481
*D. obclavata*	MFLUCC 18–0329 ^T^	MN163012	MN163010	—	—
*D. obpyriformis*	MFLUCC 17–1694 ^T^	—	MG979764	MG988415	MG988422
*D. obpyriformis*	DLUCC 0867	MG979757	MG979765	MG988416	MG988423
*D. pachyconidia*	KUMCC 21–10724 ^T^	OK310696	OK341194	OP413471	OP413477
*D. palmarum*	MFLUCC 18–1446 ^T^	MK085062	MK079663	MK087670	MK087660
*D. phangngaensis*	MFLUCC 16–0857 ^T^	MF077545	MF077556	—	MF135653
*D. phragmiticola*	GUCC 220201 ^T^	OP749887	OP749880	OP752699	OP749891
*D. phragmiticola*	GUCC 220202 ^T^	OP749888	OP749881	OP752700	OP749892
*D. rayongensis*	MFLUCC 18–0415 ^T^	MH457172	MH457137	MH463255	MH463253
*D. rayongensis*	MFLUCC 18–0417	MH457173	MH457138	MH463256	MH463254
*D. rostrata*	MFLUCC 16–0969 ^T^	MG979758	MG979766	MG988417	MG988424
*D. rostrata*	DLUCC 0885	MG979759	MG979767	—	MG988425
*D. saprophytica*	MFLUCC 18–1238 ^T^	MW286506	MW287780	MW504069	MW396651
*D. septata*	GZCC 22–0078 ^T^	ON527939	ON527947	ON533690	ON533683
*D. sinensis*	HJAUP C2044 ^T^	OP787878	OP787875	—	OP961936
*D. sichuanensis*	KUNCC 23-15519 ^T^	PP584672	PP584769	—	PP663312
*D. songkhlaensis*	MFLUCC 18–1234 ^T^	MW286482	MW287755	—	MW396642
*D. suae*	CGMCC 3.24262 ^T^	OQ874968	OQ732679	OQ870341	OR367670
*D. subtropica*	HJAUP C2528 ^T^	PQ211099	PQ211107	PQ303677	PQ303684
*D. suoluoensis*	MFLUCC 17–0224 ^T^	MF077546	MF077557	—	MF135654
*D. suoluoensis*	MFLUCC 17–0854	MF077547	MF077558	MZ945510	—
*D. tectonae*	MFLUCC 12–0291 ^T^	KX751711	KX751713	KX751708	KX751710
*D. tectonae*	MFLU 20–0262	MT232714	MT232719	—	—
*D. tectonigena*	MFLUCC 12–0292 ^T^	KX751712	KX751714	KX751709	—
***D. terrestris***	**HJAUP C2539 ^T^**	**PV448667**	**PV450538**	**—**	**PV469764**
*D. thailandica*	MFLUCC 16–0270 ^T^	MH275060	MH260292	—	MH412767
*D. thysanolaenae*	KUN–HKAS 112710	MW723057	MW879524	—	MW729783
*D. thysanolaenae*	KUN–HKAS 102247 ^T^	MK045851	MK064091	—	MK086031
*D. tropica*	GZCC 22–0076 ^T^	ON527935	ON527943	ON533687	ON533679
*D. verrucosa*	GZCC 20–0434 ^T^	MZ868771	MZ868762	MZ892983	MZ892977
***D. wuyishanensis***	**HJAUP C2515 ^T^**	**PV448666**	**PV450537**	**PV469759**	**PV469763**
*D. wuzhishanensis*	GZCC 22–0077 ^T^	ON527938	ON527946	—	ON533682
*D. xingpingensis*	KUNCC 22–12669	OQ874969	OQ732680	—	—
*D. xingpingensis*	KUNCC 22–12667 ^T^	OQ874970	OQ732681	OQ870340	OR367671
*D. xishuangbannaensis*	KUMCC 17–0290 ^T^	MH275061	MH260293	MH412754	MH412768
*D. yichunensis*	HJAUP C1065 ^T^	OQ942885	OQ942892	OQ944459	OQ944453
*D. yongxiuensis*	JAUCC 4725 ^T^	OK562388	OK562394	—	OK562406
*D. yongxiuensis*	JAUCC 4726 ^T^	OK562389	OK562395	—	OK562407
*D. yunjushanensis*	JAUCC 4723 ^T^	OK562392	OK562398	—	OK562410
*D. yunjushanensis*	JAUCC 4724 ^T^	OK562393	OK562399	—	OK562411
*D. yunnansis*	MFLUCC 20–0153 ^T^	MW081541	MW081546	MW151861	MW084995
***D. zhejiangensis***	**HJAUP C2588 ^T^**	**PV448668**	**PV450539**	**—**	**PV469765**
*Fluminicola saprophytica*	MFLUCC 15–0976 ^T^	MF374358	MF374367	MF370954	MF370956
*Myrmecridium banksiae*	CBS 132536 ^T^	JX069871	JX069855	—	—
*M. schulzeri*	CBS 100.54	EU041769	EU041826	—	—
*Papulosa amerospora*	AFTOL–ID 748	—	DQ470950	DQ470901	DQ471069
*Pleurophragmium bambusinum*	MFLUCC 12–0850	KU940161	KU863149	—	KU940213
*Pseudostanjehughesia aquitropica*	MFLUCC 16–0569 ^T^	MF077548	MF077559	—	MF135655
*P. lignicol*	MFLUCC 15–0352 ^T^	MK828643	MK849787	MN124534	MN194047
*Wongia griffinii*	DAR 80512 ^T^	KU850473	KU850471	—	—

**Table 3:** The *Helminthosporium* species and relevant species with their corresponding GenBank accession numbers used in the phylogenetic analyses of this study. The ex-type cultures are indicated using “^T^” after strain numbers; “—” stands for no sequence data in GenBank.

Taxon	Strain Number	GenBank Accession Numbers				
		ITS	LSU	*RPB2*	SSU	*TEF1*
*Byssothecium circinans*	CBS 675.92	OM337536	GU205217	DQ767646	GU205235	GU349061
*Corynespora cassiicola*	CBS 100822	—	GU301808	GU371742	GU296144	GU349052
*C. smithii*	L120	KY984297	KY984297	KY984361	—	KY984435
*C. smithii*	L130	KY984298	KY984298	KY984362	KY984419	KY984436
*Cyclothyriella rubronotata*	CBS 121892	KX650541	KX650541	KX650571	—	KX650516
*C. rubronotata*	CBS 141486 ^T^	KX650544	KX650544	KX650574	KX650507	KX650519
*Helminthosporium aquaticum*	MFLUCC 15–0357 ^T^	KU697302	KU697306	—	KU697310	—
*H. austriacum*	CBS 139924 ^T^	KY984301	KY984301	KY984365	KY984420	KY984437
*H. austriacum*	CBS 142388	KY984303	KY984303	KY984367	—	KY984439
*H. caespitosum*	CBS 484.77 ^T^	JQ044429	JQ044448	KY984370	KY984421	KY984440
*H. caespitosum*	L141	KY984305	KY984305	KY984368	—	—
*H. chengduense*	UESTC 22.0024 ^T^	ON557751	ON557745	ON563073	ON557757	ON600598
*H. chengduense*	UESTC 22.0025	ON557750	ON557744	ON563072	ON557756	ON600597
*H. chiangraiense*	MFLUCC 21–0087 ^T^	MZ538504	MZ538538	—	—	—
*H. chinense*	CGMCC 3.23570 ^T^	ON557754	ON557748	—	ON557760	ON600601
*H. chlorophorae*	BRIP 14521	AF120259	—	—	—	—
*H. dalbergiae*	MAFF 243853	LC014555	AB807521	—	AB797231	AB808497
*H. endiandrae*	CBS 138902 ^T^	KP004450	KP004478	—	—	—
*H. erythrinicola*	CBS 145569 ^T^	NR_165563	MK876432	MK876486	—	—
*H. filamentosum*	UESTCC 24.0132 ^T^	PP835322	PP835316	PP844886	PP835319	PP844888
***H. ganzhouense***	**HJAUP C1086 ^T^**	**PV448665**	**PV450532**	**PP501323**	**PV450540**	**PP501325**
*H. genistae*	CBS 139922	KY984309	KY984309	KY984373	KY984423	—
*H. genistae*	CBS 142597 ^T^	KY984310	KY984310	KY984374	—	—
*H. genistae*	CBS 139927	KY984311	KY984311	KY984375	—	—
*H. genistae*	CBS 139928	KY984312	KY984312	KY984376	—	—
*H. genistae*	CBS 139929	KY984315	KY984315	KY984379	—	—
*H. genistae*	CBS 139930	KY984316	KY984316	KY984380	—	—
*H. guanshanense*	HJAUP C1022 ^T^	OQ172249	OQ172239	OQ234978	OQ172247	OQ256247
*H. guizhouense*	GUCC24–0011 ^T^	PP915799	PP949847	PP947940	PP949912	—
*H. guizhouense*	HKAS 130313	PP981907	PP981904	—	PP977159	PQ041228
*H. hispanicum*	CBS 136917 ^T^	KY984318	KY984318	KY984381	KY984424	KY984441
*H. italicum*	MFLUCC 17–0241 ^T^	KY797638	KY815015	—	—	KY815021
***H. jiangxiense***	**HJAUP C1325 ^T^**	**PV448662**	**PV450534**	**–**	**PV450542**	**PV469760**
*H. jiulianshanense*	HJAUP C1057 ^T^	OQ172245	OQ172253	OQ234979	—	—
*H. juglandinum*	CBS 136912	KY984319	KY984319	KY984382	—	KY984442
*H. juglandinum*	CBS 136913	KY984320	KY984320	—	—	—
*H. juglandinum*	CBS 136922 ^T^	KY984321	KY984321	KY984383	—	KY984443
*H. juglandinum*	CBS 136911	KY984322	KY984322	—	KY984425	—
*H. leucadendri*	CBS 135133 ^T^	KF251150	KF251654	KF252159	—	KF253110
*H. lignicola*	MFLUCC 22–0118 ^T^	—	OP740252	OP757656	OP740253	OP757657
*H. livistonae*	CBS144413 ^T^	NR_160348	NG_064539	—	—	—
*H. magnisporum*	MAFF 239278 ^T^	AB811452	AB807522	—	AB797232	AB808498
*H. massarinum*	JCM 13094	AB809628	AB807523	—	AB797233	AB808499
*H. massarinum*	CBS 139690 ^T^	AB809629	AB807524	—	AB797234	AB808500
*H. meilingense*	HJAUP C1076 ^T^	OQ172244	OQ172238	OQ234980	OQ172246	OQ234981
*H. microsorum*	L94	KY984327	KY984327	KY984388	KY984426	KY984446
*H. microsorum*	L95	KY984328	KY984328	KY984389	—	KY984447
*H. microsorum*	CBS 136910 ^T^	KY984329	KY984329	KY984390	KY984427	KY984448
*H. nabanhense*	HJAUP C2054 ^T^	OP555394	OP555398	—	OP555400	OP961931
*H. nanjingensis*	HHAUF020380	KF192322	—	—	—	—
*H. oligosporum*	CBS 136908	KY984332	KY984332	KY984393	KY984428	KY984450
*H. oligosporum*	CBS 136909 ^T^	KY984333	KY984333	KY984394	—	KY984451
*H. paraoligosporum*	EI–418	ON206009	OP888100	—	—	—
*H. puerensis*	KUNCC 24–18347 ^T^	OR428391	OR428413	OR515515	OR428371	OR509745
*H. pini*	HKAS 135177 ^T^	PP835323	PP835317	PP844887	PP835320	PP844889
*H. quercinum*	CBS 112393	KY984334	KY984334	KY984395	—	KY984452
*H. quercinum*	CBS 136915	KY984336	KY984336	KY984397	—	—
*H. quercinum*	CBS 136921 ^T^	KY984339	KY984339	KY984400	KY984429	KY984453
***H. saprophyticum***	**HJAUP C2572 ^T^**	**PV448663**	**PV450536**	**PV469758**	**PV450544**	**PV469761**
***H. saprophyticum***	**HJAUP C2573**	**PV448664**	**PV450535**	**–**	**PV450543**	**PV469762**
*H. shangrilaense*	KUNCC 22–12540 ^T^	OP767128	OP767126	—	OP767127	—
*H. sinense*	HJAUP C2121 ^T^	OP555393	OP555397	—	OP555399	OP961932
*H. solani*	CBS 365.75	KY984341	KY984341	KY984402	KY984430	KY984455
*H. solani*	CBS 640.85	KY984342	KY984342	KY984403	—	—
*H. submersum*	MFLUCC 16–1360 ^T^	—	MG098787	—	MG098796	MG098586
*H. submersum*	MFLUCC 16–1290	MG098780	MG098788	MG098592	MG098797	MG098587
*H. syzygii*	CBS 145570 ^T^	NR_165564	MK876433	MK876487	—	—
*H. thailandicum*	MFLU 24-0275 ^T^	PQ325264	PQ578298	—	PQ651973	—
*H. tiliae*	CBS 136906	KY984344	KY984344	KY984405	—	—
*H. tiliae*	CBS 136907 ^T^	KY984345	KY984345	KY984406	KY984431	KY984457
*H. velutinum*	H 4626	LC014556	AB807530	—	AB797240	AB808505
*H. velutinum*	CBS 136924	KY984347	KY984347	KY984408	—	KY984458
*H. velutinum*	CBS 139923 ^T^	KY984352	KY984352	KY984413	KY984432	KY984463
*H. velutinum*	L98	KY984359	KY984359	KY984417	KY984433	KY984466
***H. velutinum***	**HJAUP C1289**	**PV448661**	**PV450533**	**PP501324**	**PV450541**	**PP501326**
*H. yunnanense*	HJAUP C2071 ^T^	OP555395	OP555396	OP961934	OP555392	OP961933
*Massarina cisti*	CBS 266.62 ^T^	LC014568	AB807539	—	AB797249	AB808514
*M. eburnea*	CBS 473.64	AF383959	GU301840	GU371732	AF164367	GU349040
*M. eburnea*	CBS 139697	LC014569	AB521735	—	AB521718	AB808517
*Periconia byssoides*	MAFF243872	LC014581	AB807570	—	AB797280	AB808546
*P. digitata*	CBS 510.77	LC014584	AB807561	—	AB797271	AB808537
*P. macrospinosa*	CBS 135663	KP183999	KP184038	—	KP184080	—
*P. pseudodigitata*	CBS139699	NR_153490	NG_059396	—	NG_064850	AB808540
*Pseudosplanchnonema phorcioides*	CBS 122935	KY984360	KY984360	KY984418	KY984434	KY984467
*Stagonospora paludosa*	CBS 135088	KF251257	KF251760	KF252262	—	KF253207
*S. perfecta*	MAFF 239609	AB809642	AB807579	—	AB797289	AB808555
*S. pseudoperfecta*	CBS 120236 ^T^	AB809641	AB807577	—	AB797287	AB808553
*S. tainanensis*	MAFF 243860	AB809643	AB807580	—	AB797290	AB808556

## Data Availability

All sequences generated in this study were submitted to GenBank.
